# Silent Phaeochromocytoma in a Young Normotensive Woman

**DOI:** 10.7759/cureus.110981

**Published:** 2026-06-16

**Authors:** Ashakumari Badiani, Stephanie Siew, Mustafa Al-Ansari

**Affiliations:** 1 Endocrinology, Hillingdon Hospital, London, GBR

**Keywords:** adrenal incidentalomas, adrenal pheochromocytoma, neuroendocrine tumour, normotension, plasma metanephrines

## Abstract

Phaeochromocytomas typically present with episodic palpitations, sweating and headaches, often with paroxysmal hypertension. In contrast, a smaller proportion are detected incidentally and may be normotensive, creating diagnostic challenges. We present the case of a 28-year-old British Indian woman who was incidentally found to have a right adrenal mass on computed tomography (CT) performed following an occupational abdominal injury. She was normotensive at the time and did not describe symptoms of classical catecholamine excess. However, she reported intermittent anxiety-like symptoms, which she attributed to work-related stress. Contrast-enhanced CT of the abdomen and pelvis revealed a 4cm right adrenal lesion with high attenuation of ~90 Hounsfield units (HU) and a heterogeneous appearance, raising suspicion of a non-adenomatous adrenal mass. Biochemical analysis demonstrated significantly raised plasma metanephrines on two separate occasions, consistent with phaeochromocytoma. Following multidisciplinary team (MDT) discussion, she was commenced on phenoxybenzamine and subsequently underwent an elective laparoscopic right adrenalectomy. Histology confirmed a completely excised phaeochromocytoma with low-risk features (Pheochromocytoma of the Adrenal Gland Scaled Score (PASS) 2; Ki-67 <2%) and retained succinate dehydrogenase subunit B (SDHB) staining. Post-operatively, she remained normotensive, and follow-up plasma metanephrines were normal, with no evidence of residual disease. This case illustrates the diagnostic difficulties that can arise in normotensive, minimally symptomatic patients, and highlights the importance of systematic MDT evaluation of adrenal incidentalomas and cautious peri-operative planning, even in “silent” disease.

## Introduction

Background

Phaeochromocytomas are rare neuroendocrine tumours arising from adrenal medullary chromaffin cells and are characterised by catecholamine excess [1]. If undiagnosed, they may be associated with serious cardiovascular complications related to catecholamine release [2]. They are traditionally associated with headache, palpitations, diaphoresis and paroxysmal or sustained hypertension [1,3]. However, their clinical presentation is variable, and contemporary series suggest that a substantial proportion are now detected incidentally on cross-sectional imaging performed for unrelated indications [3,4]. As a result, some patients may be minimally symptomatic or normotensive at the time of diagnosis [3,5]. This can hinder timely recognition, particularly when symptoms are absent, intermittent or non-specific. 

This case is of particular interest as it represents a challenging presentation: a biochemically active yet clinically “silent” phaeochromocytoma presenting as an adrenal incidentaloma in a normotensive patient. It highlights the importance of a structured approach to adrenal incidentaloma work-up that does not solely rely on classical signs and symptoms. Current adrenal incidentaloma guidance recommends that adrenal masses be assessed for both malignant potential and hormone excess, with imaging characteristics used to guide further evaluation [6]. In particular, homogeneous adrenal lesions measuring ≤10 Hounsfield units (HU) on unenhanced computed tomography (CT) are considered consistent with benign lipid-rich adenomas; therefore, lesions that do not meet these benign imaging criteria require further assessment, including biochemical evaluation for phaeochromocytoma where appropriate [6]. Lastly, confirmed functional phaeochromocytoma requires peri-operative preparation with alpha-adrenergic blockade, together with high-sodium intake and fluid intake, to reduce peri-operative cardiovascular complications and prevent post-operative hypotension [1].

## Case presentation

A 28-year-old Indian woman presented to the emergency department in July 2023 following a workplace injury. A CT scan performed during her assessment incidentally identified a 4 cm high-attenuating right adrenal lesion. The radiological differentials included haemorrhage into a pre-existing adrenal nodule, a new adrenal haematoma, or a pre-existing adrenal mass without acute haemorrhage. At subsequent endocrine clinic follow-up, she reported intermittent anxiety attacks that had been present prior to the workplace incident, which she attributed to studies and work-related stress. She reported no other classical symptoms suggestive of phaeochromocytoma, and blood pressures recorded across clinic appointments remained within the normotensive range. She also described weight gain of approximately 13 kg over several years with abdominal striae, long-standing facial acne, mild hirsutism and menstrual irregularity. Her past medical history included a previous proximal subclavian artery thrombosis (resolved), for which she was taking aspirin. Family history included type 2 diabetes mellitus and hypertension. She was a non-smoker with no alcohol consumption. Examination demonstrated normal vital signs, including a blood pressure of 122/76 mmHg, with a BMI of 32 kg/m². On clinical examination, mild hirsutism was present, without overt Cushingoid features.

Investigation

The initial CT abdomen and pelvis with contrast (Figure 1) demonstrated a well-circumscribed right adrenal mass measuring 4 cm with high attenuation (~90 HU). Subsequent MRI (Figure 2) demonstrated a heterogeneous right adrenal lesion (~45 × 36 mm) on T2-weighted sequences, without signal loss on out-of-phase imaging, with heterogeneous enhancement. The radiological differential included phaeochromocytoma and primary adrenal malignancy. 

**Figure 1:**
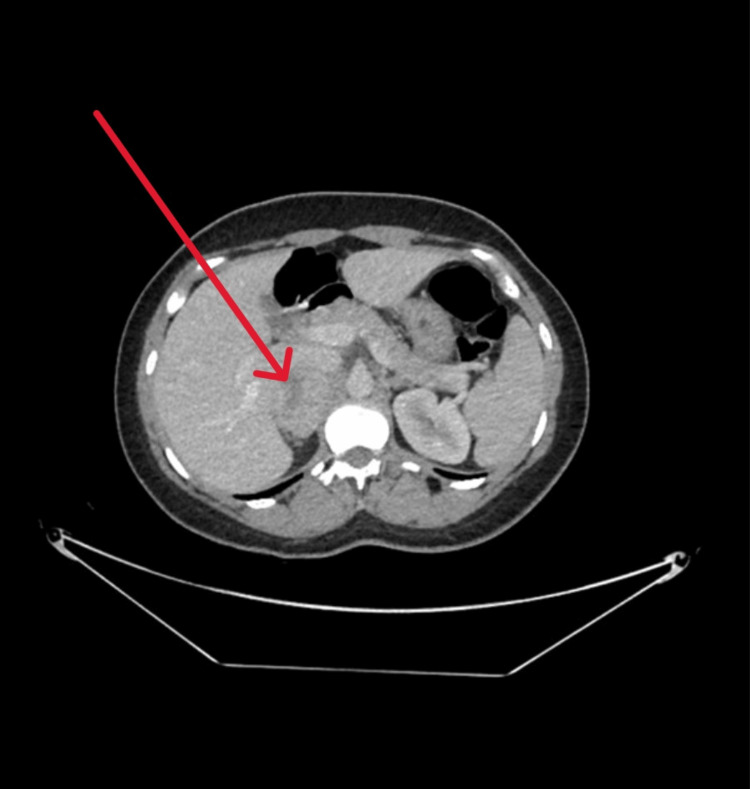
Contrast-enhanced CT abdomen and pelvis study (portal venous phase); axial image demonstrating a 4 cm right adrenal lesion

**Figure 2:**
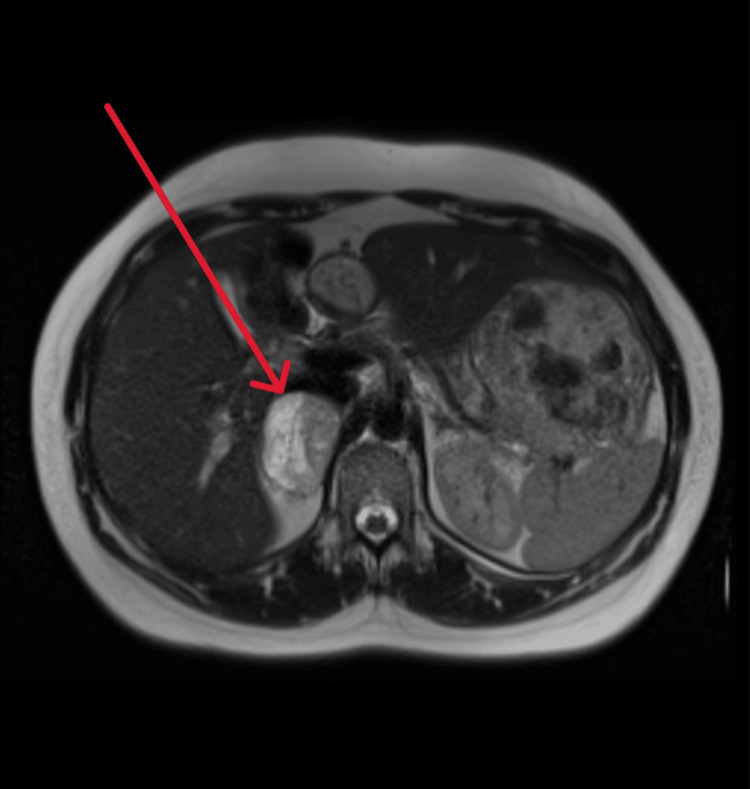
Non-contrast MRI adrenal study; axial T2 image demonstrating the right adrenal lesion. The lesion is of predominantly high T2 signal, a sign typically seen with phaeochromocytomas

Further biochemical work-up (presented in Table 1) revealed markedly elevated plasma metanephrines on repeat testing (normetadrenaline 2733 to 5616 pmol/L; metadrenaline 3488 to 4885 pmol/L; 3-methoxytyramine <85 pmol/L), supporting a diagnosis of phaeochromocytoma.

**Table 1:** Biochemistry and baseline blood tests during adrenal incidentaloma work-up eGFR: estimated glomerular filtration rate, LDL: low-density lipoprotein, DHEA-S: dehydroepiandrosterone sulfate

	Reference range	Reported result	Interpretation
Haemoglobin (Hb), g/L	114-150	109	Low
eGFR, mL/min/1.73m²	>90	>90	Normal
HbA1c, mmol/mol	20-41	38	Normal
Total cholesterol, mmol/L	<5.0	5.9	High
LDL cholesterol, mmol/L	<3.0	4.3	High
Triglycerides (TGs), mmol/L	<1.70	1.1	Normal
1 mg overnight dexamethasone suppression test (09:00 cortisol), nmol/L	<50	33	Normal
Normetadrenaline (plasma), pmol/L	<1180	2733 (sample 1)	High
Normetadrenaline (plasma), pmol/L	<1180	5616 (sample 2; peak)	High
Metadrenaline (plasma), pmol/L	<510	3488 (sample 1)	High
Metadrenaline (plasma), pmol/L	<510	4885 (sample 2; peak)	High
3-Methoxytyramine (plasma), pmol/L	<180	<85	Normal
DHEA-S, µmol/L	1.2–8	3.6	Normal
17-Hydroxyprogesterone, nmol/L	Adult females (16+): Follicular 0.6–4.0; Luteal 1.0–6.0	<2	Normal
Testosterone, nmol/L	≤2.0	0.9	Normal

Although the patient reported features suggestive of hypercortisolism, screening was reassuring, with an appropriate cortisol suppression with 1 mg overnight dexamethasone suppression test (cortisol 33 nmol/L). The aldosterone-renin ratio was also normal. In addition, there was no biochemical evidence of androgen excess (DHEA-S 3.6 μmol/L, 17-hydroxyprogesterone <2 nmol/L, testosterone 0.9 nmol/L).

The case was subsequently reviewed at the adrenal multidisciplinary team (MDT) meeting.

Treatment

Following the adrenal MDT discussion, the patient was commenced on pre-operative alpha-adrenergic blockade and dietary measures. Oral phenoxybenzamine was initiated at 10 mg once daily and then up-titrated to 10 mg twice daily according to clinical response, with advice to increase fluid intake and follow a high-salt diet (approximately 4 g/day). She subsequently underwent an elective right laparoscopic adrenalectomy in March 2024. 

Outcome and follow-up

Following surgery, the patient made a good recovery and has remained normotensive to date. Histopathology confirmed a completely excised right adrenal pheochromocytoma. The Pheochromocytoma of the Adrenal Gland Scaled Score (PASS) was 2 (cellular spindling). On immunohistochemistry, tumour cells showed diffuse strong expression of synaptophysin, chromogranin, and GATA-3, with retained succinate dehydrogenase subunit B (SDHB) staining. The tumour was negative for SOX-10, inhibin, and Melan A; S100 was non-contributory. The Ki-67 proliferation index was <2%, supporting low proliferative activity. 

## Discussion

Diagnostic difficulties

This case highlights a diagnostic pitfall that is increasingly relevant in the field of endocrinology: the incidental detection of a phaeochromocytoma in a normotensive young patient with minimal symptoms. Hypertension remains a useful diagnostic clue, but it is not universal, and reliance on typical presentations can delay recognition in patients with non-specific symptoms. In our patient, intermittent anxiety-related symptoms were present. However, these were attributed to occupational stressors, lowering clinical suspicion until further endocrine evaluation was undertaken. 

Incidental detection and normotensive disease 

Current adrenal incidentaloma guidelines reflect the increasing detection of adrenal lesions on imaging performed for unrelated indications [6]. The 2023 European Society of Endocrinology (ESE) guidance emphasises risk stratification based on imaging characteristics, with endocrine evaluation tailored to the malignancy risk and the likelihood of hormonal hypersecretion [6]. Notably, normal blood pressure does not exclude clinically significant catecholamine excess. Recent studies suggest that a substantial proportion of phaeochromocytomas present in normotensive patients, accounting for approximately 50% of reported cases [7]. This shifting epidemiology has resulted in “silent” presentations being more frequently encountered and highlights the importance of structured diagnostic pathways to prevent missed or delayed diagnoses. 

Brief literature reviews 

The literature increasingly reflects a transition from symptom-based diagnosis to a more incidental detection of phaeochromocytomas, with a parallel recognition that classical features, including hypertension, may be absent [3,4,8]. Furthermore, studies comparing normotensive and hypertensive phaeochromocytoma cohorts highlighted that patients with normal blood pressure should not be equated with low risk [7,9]. Peri-operative haemodynamic instability and post-operative complications may still occur, reinforcing the need for careful peri-operative planning regardless of an individual’s baseline blood pressure [9]. Overall, the evidence supports a pragmatic approach: clinicians should maintain diagnostic vigilance for functional tumours in the context of atypical imaging, even when “red flag” symptoms are not present.

Imaging cues and incidentaloma work-up 

In our case, the imaging phenotype was the main driver for further hormonal evaluation. The lesion was high attenuating (~90 HU) and heterogeneous, and MRI did not demonstrate features consistent with a benign lipid-rich adenoma. Current ESE guidance notes that an unenhanced CT attenuation of ≤10 HU makes phaeochromocytoma very unlikely, whereas lesions with higher attenuation or indeterminate characteristics warrant further investigation [6]. This case therefore highlights the practical importance of a radiology-led incidentaloma pathway: atypical imaging features should prompt endocrine evaluation even when clinical suspicion is low. 

Biochemical confirmation 

For suspected phaeochromocytoma, the Endocrine Society guideline recommends plasma-free or urinary fractionated metanephrines as first-line biochemical tests, with attention to pre-analytical factors and repeat testing where results are equivocal [1]. In this case, plasma metanephrines were markedly elevated on repeat sampling, making a false-positive result unlikely and supporting definitive management in the context of an indeterminate adrenal lesion. In minimally symptomatic patients, biochemical confirmation is of significant relevance, as clinicians may otherwise hesitate to refer for surgical assessment.

Peri-operative preparation despite normotension 

Alpha-adrenergic blockade is recommended in current guidelines for functional phaeochromocytomas to reduce the peri-operative risk of catecholamine-related complications, alongside salt and fluid loading to minimise the risk of post-operative hypotension [1]. However, the evidence base for the role of alpha-blockade remains controversial: meta-analyses of observational studies show inconsistent benefit for intraoperative haemodynamic outcomes, and the certainty of the evidence is low [10,11]. Despite this, practice largely remains guideline-adherent, given the unpredictable nature of catecholamine surges and the limited evidence from randomised trials [1,10,11]. In our patient, orthostatic symptoms during phenoxybenzamine titration were consistent with effective alpha-blockade, whilst also highlighting the need for careful dose optimisation and volume repletion. 

Histology, risk stratification and follow-up

Histology confirmed complete excision of the adrenal lesion with low proliferative activity, with a Ki-67 index of <2%, and a low PASS of 2. PASS was originally developed to aid risk stratification, with scores ≥4 suggested to indicate a higher likelihood of aggressive behaviour [12]. However, subsequent studies show variable reproducibility and significant inter-observer variation; therefore, PASS should not be used in isolation to guide follow-up [13]. Other grading approaches, such as Grading system for Adrenal Pheochromocytoma and Paraganglioma (GAPP; which incorporates Ki-67 and catecholamine phenotype), aim to reduce subjectivity, although no system reliably predicts malignant behaviour and metastasis remains the defining criterion [14]. Retained SDHB staining reduced the likelihood of an SDH-deficient tumour phenotype and, together with negative SDHB/SDHD germline testing documented in follow-up, supported a lower-risk profile. Nonetheless, long-term biochemical surveillance remains appropriate given the possibility of late recurrence. 

Learning points

This case reinforces several key considerations in the diagnosis and peri-operative management of phaeochromocytoma presenting as an adrenal incidentaloma. Although phaeochromocytomas typically present with paroxysmal hypertension, headache, palpitations and diaphoresis, clinically significant catecholamine-secreting tumours may also occur in normotensive or minimally symptomatic patients [1,3,7]. In such cases, non-specific symptoms may be incorrectly attributed to stress or anxiety, potentially delaying further biochemical evaluation. Radiological assessment therefore plays an important role in guiding subsequent investigation. Indeterminate or high-attenuation adrenal lesions, such as those measuring greater than 10 HU on unenhanced CT, warrant biochemical testing to exclude functional tumours, including phaeochromocytoma, cortisol excess and primary aldosteronism where clinically appropriate, alongside assessment for malignancy [6]. Repeatedly and markedly elevated plasma metanephrines strongly support the diagnosis of phaeochromocytoma and should prompt multidisciplinary discussion and surgical referral [1]. Furthermore, pre-operative optimisation remains a key aspect of management, with alpha-blockade and salt and fluid loading used to reduce the risk of peri-operative catecholamine-related complications and post-operative hypotension [1]. Following resection, histological markers such as PASS and Ki-67 may aid risk stratification; however, long-term follow-up remains essential to detect residual or recurrent disease [12-14].

## Conclusions

This case demonstrates a clinically important presentation of phaeochromocytoma: an incidentally detected, biochemically active adrenal tumour in a young patient who remained persistently normotensive, with symptoms that were easily misattributed to anxiety or stress. It highlights that normotension and absence of classical symptoms do not exclude clinically significant catecholamine excess. A structured approach to adrenal incidentaloma evaluation can reduce missed or delayed diagnoses. High-attenuation or heterogeneous lesions should prompt timely biochemical testing and multidisciplinary review to support safe surgical planning. Finally, even in normotensive patients, guideline-consistent peri-operative optimisation with alpha-blockade and salt and fluid loading remains essential to mitigate haemodynamic instability, and long-term biochemical surveillance is warranted despite reassuring low-risk histology. 
